# Supplementary material for: Illuminating the lineage-specific diversification of resin glycoside acylsugars in the morning glory (Convolvulaceae) family using computational metabolomics
Source: Hortic Res. 2022 Feb 4;9:uhab079. doi: 10.1093/hr/uhab079 (PMC8825387; doi:10.1093/hr/uhab079)
Supplement: Web_Material_uhab079 [file web_material_uhab079.zip › SuppFile3_LCMSparameters.docx.docx]

***Supplementary File 3: Description of LC-MS methods used in this study***

**UHPLC method**

Instrument: Thermo Fisher Dionex UltiMate 3000 UHPLC

Column Oven: 40.0°C

Solvent A: Water with 0.1% formic acid

Solvent B: Acetonitrile

Flow Rate: 0.6 ml/min

Injection Volume (µl): 2.00

| Time (Min) | %B |
| --- | --- |
| 0.00 | 25.0 |
| 0.10 | 25.0 |
| 5.60 | 100.0 |
| 6.60 | 100.0 |
| 6.61 | 25.0 |
| 7.51 | 25.0 |

**MS/MS method**

Instument: Thermo Fisher Q Exactive Hybrid Quadrupole-Orbitrap Mass Spectrometer

Polarity: negative

**Full MS**

Resolution: 70,000

AGC target: 5e6

Maximum IT: 30 ms

Scan range: 500 to 2000 m/z

**dd-MS²**

Resolution: 17,500

AGC target: 5e5

Maximum IT: 110 ms

Loop count: 10

TopN: 10

Isolation window: 1.5 m/z

Scan range: 200 to 2000 m/z

NCE: 20, 40

Minimum AGC target: 5.00e3

Intensity threshold: 4.5e4

Dynamic exclusion: 7.0 s

**Source**

Spray Voltage (-): 2500.00

Capillary Temperature (-): 380.00

Sheath Gas (-): 70.00

Aux Gas (-): 20.00

Max Spray Current (-): 100.00

Probe Heater Temp. (-): 400.00

S-Lens RF Level: 75.00

Ion Source: HESI

**MS-DIAL parameters:**

#Project

MS1 Data type Profile

MS2 Data type Profile

Ion mode Negative

Target Metablomics

Mode ddMSMS

#Data collection parameters

Retention time begin 0

Retention time end 7

Mass range begin 0

Mass range end 2000

MS2 mass range begin 0

MS2 mass range end 2000

#Centroid parameters

MS1 tolerance 0.005

MS2 tolerance 0.01

#Isotope recognition

Maximum charged number 2

#Data processing

Number of threads 1

#Peak detection parameters

Smoothing method LinearWeightedMovingAverage

Smoothing level 2

Minimum peak width 3

Minimum peak height 10000

#Peak spotting parameters

Mass slice width 0.1

Exclusion mass list (mass & tolerance)

#Deconvolution parameters

Sigma window value 0.5

MS2Dec amplitude cut off 0

Exclude after precursor True

Keep isotope until 0.5

Keep original precursor isotopes False

#MSP file and MS/MS identification setting

MSP file

Retention time tolerance 0

Accurate mass tolerance (MS1) 0.1

Accurate mass tolerance (MS2) 0.1

Identification score cut off 80

Using retention time for scoring True

Using retention time for filtering False

#Text file and post identification (retention time and accurate mass based) setting

Text file

Retention time tolerance 0.1

Accurate mass tolerance 0.01

Identification score cut off 85

#Advanced setting for identification

Relative abundance cut off 0

Top candidate report True

#Adduct ion setting

[M-H]-

[M-H2O-H]-

[M+FA-H]-

#Alignment parameters setting

Reference file <filename>.abf

Retention time tolerance 0.1

MS1 tolerance 0.05

Retention time factor 0.5

MS1 factor 0.5

Peak count filter 0

N% detected in at least one group 0

Remove feature based on peak height fold-change True

Sample max / blank average 5

Sample average / blank average 5

Keep identified and annotated metabolites True

Keep removable features and assign the tag for checking True

Gap filling by compulsion False

#Tracking of isotope labels

Tracking of isotopic labels FALSE

#Ion mobility

Ion mobility data FALSE
