# Supplementary material for: Illuminating the lineage-specific diversification of resin glycoside acylsugars in the morning glory (Convolvulaceae) family using computational metabolomics
Source: Hortic Res. 2022 Feb 4;9:uhab079. doi: 10.1093/hr/uhab079 (PMC8825387; doi:10.1093/hr/uhab079)
Supplement: Web_Material_uhab079 [file web_material_uhab079.zip › Supplementary Figures Combined.pdf]

Supplementary Figure 1

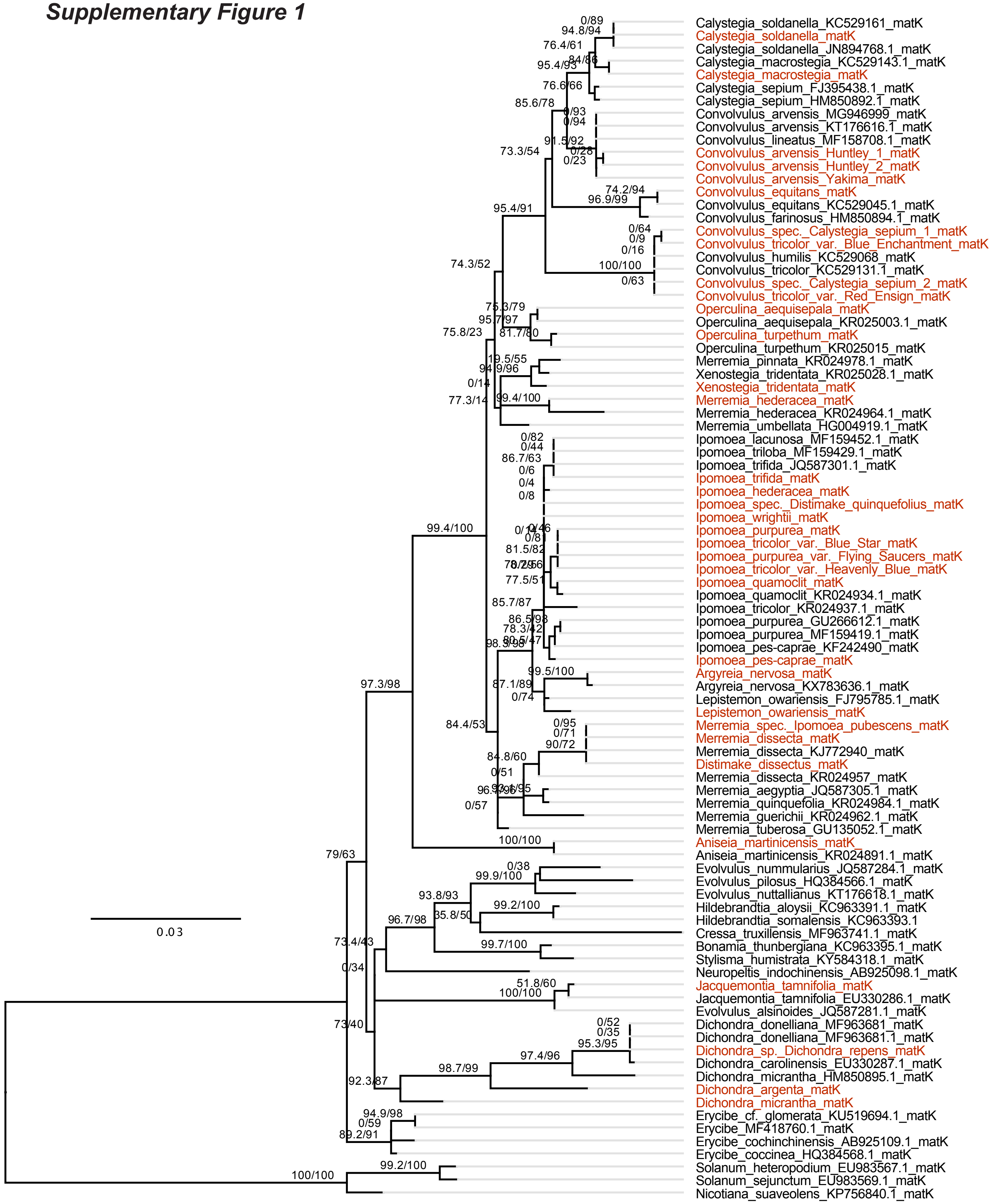

Supplementary Figure 1: matK phylogeny of selected species. matK sequences were obtained from Sanger sequencing of the tested species (red) as well as from the NCBI database (black). The tree was optained with IQ-TREE using the SH-like approximate likelihood ratio test (SH-aLRT,1000 replicates) and 1000 standard non-parametric bootstrap replicates. Branch support values are SH-aL-RT/bootstrap support. The tree is rooted on the brach leading to the included Solanaceae species.

Supplementary Fig. 2

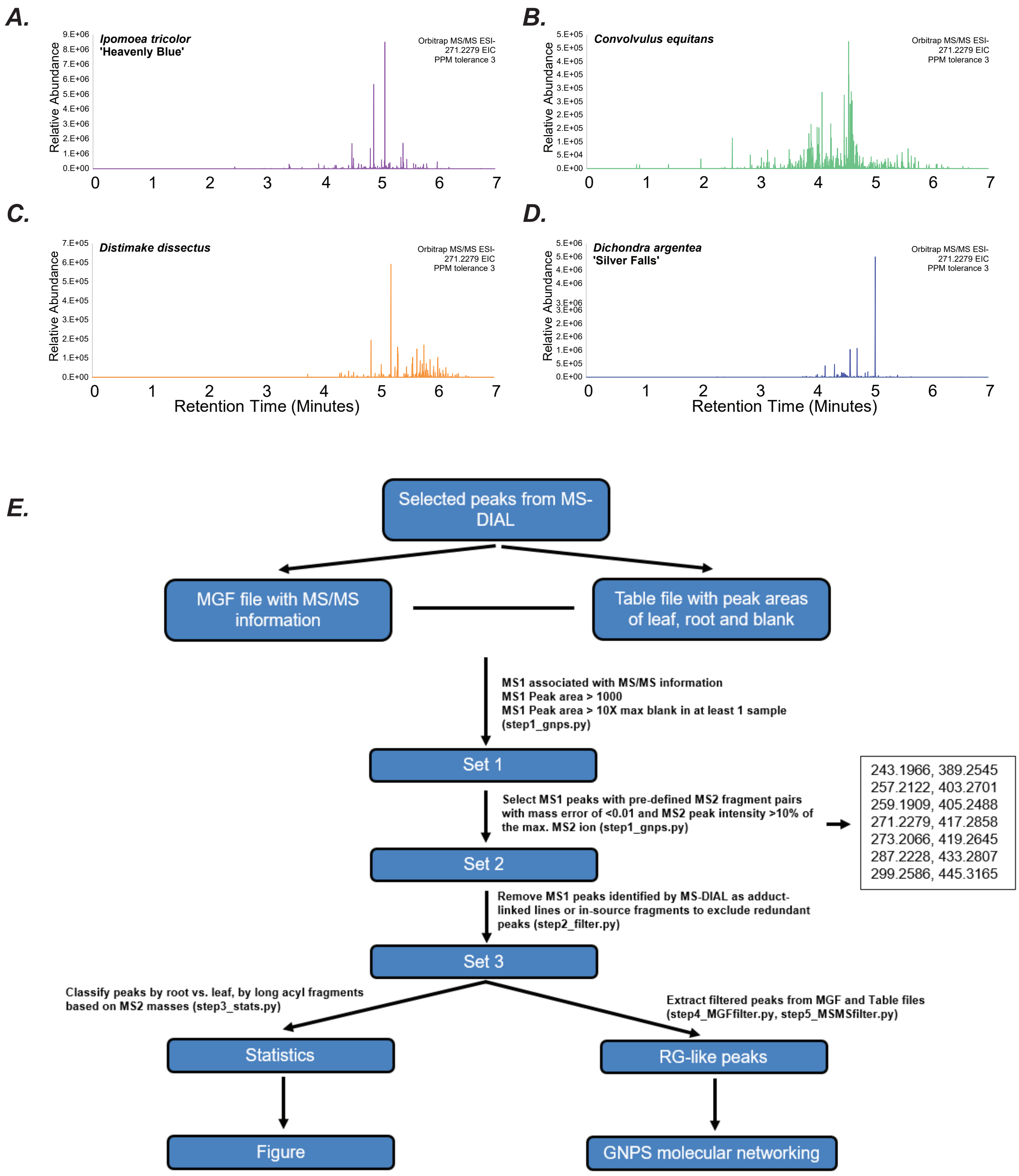

Supplementary Fig. 2: Resin glycoside diversity. (A-D) Extracted ion chromatograms of m/z 271.2279 -- corresponding to the mass of jalapinolic acid -- from four Convolvulaceae species. (E) Computational pipeline used for RG detection. An example of the RG signature pairs is shown, out of 30 pairs (10 acyl chains x 3 sugar types) used for actual analysis.

Supplementary Figure 3

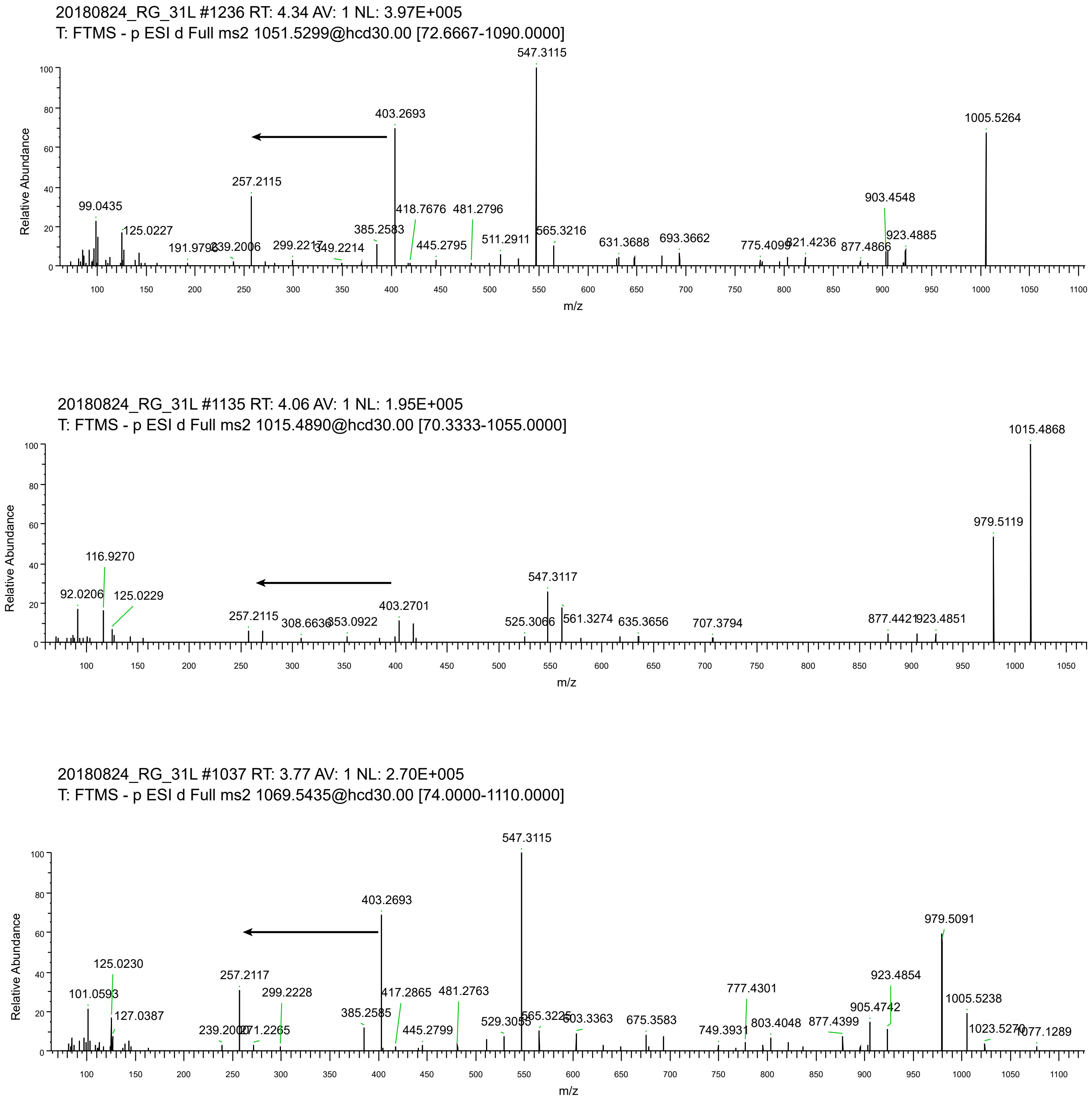

Supplementary Figure 3: Paired MS/MS fragments for the 257 fragment. Arrows limit peaks corresponding to the hydroxyacyl chain (smaller mass peak) and the hydroxyacyl chain+sugar (larger mass peak).

Supplementary Figure 4

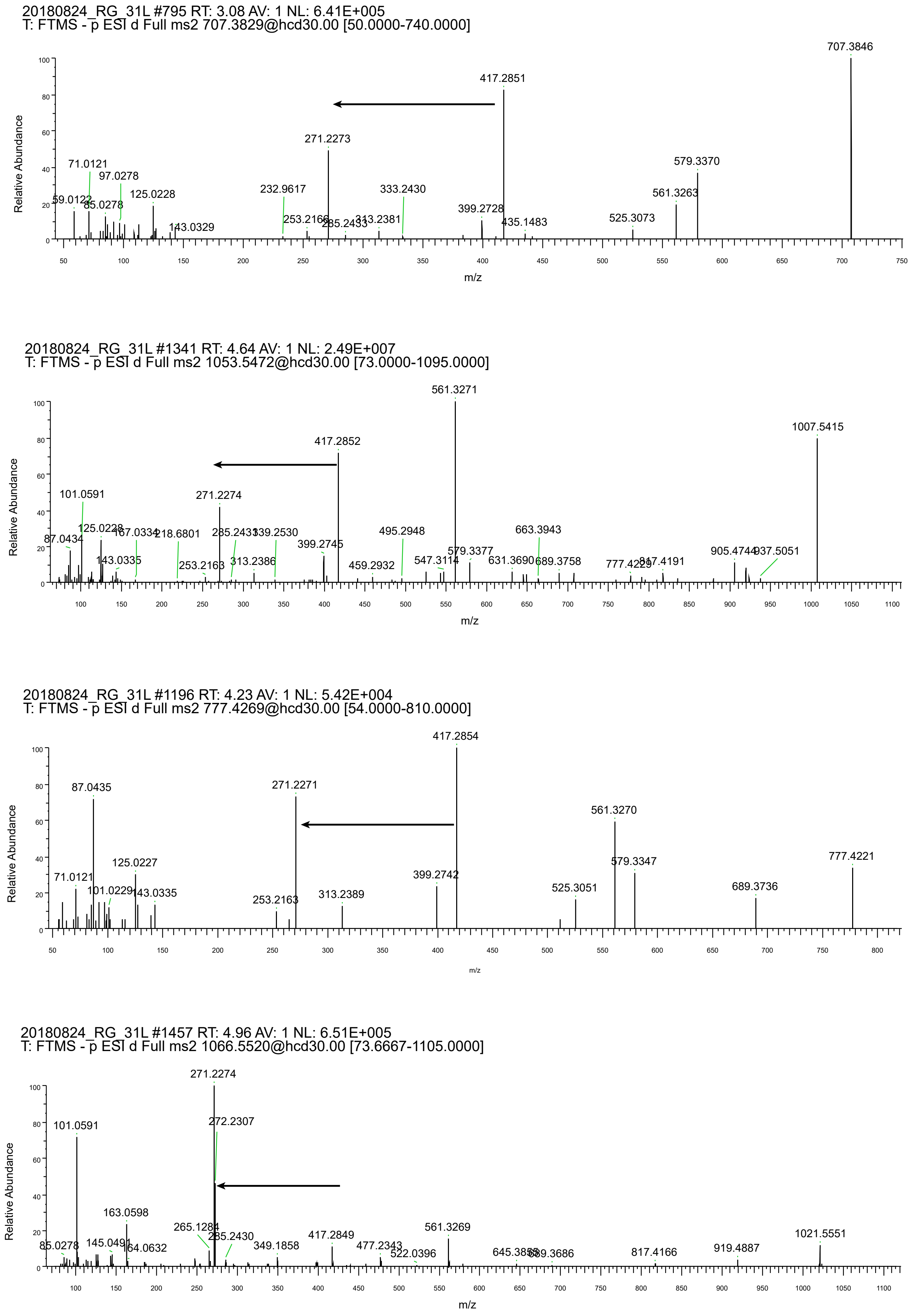

Supplementary Figure 4: Paired MS/MS fragments for the 271 fragment. Arrows limit peaks corresponding to the hydroxyacyl chain (smaller mass peak) and the hydroxyacyl chain+sugar (larger mass peak).

Supplementary Figure 5

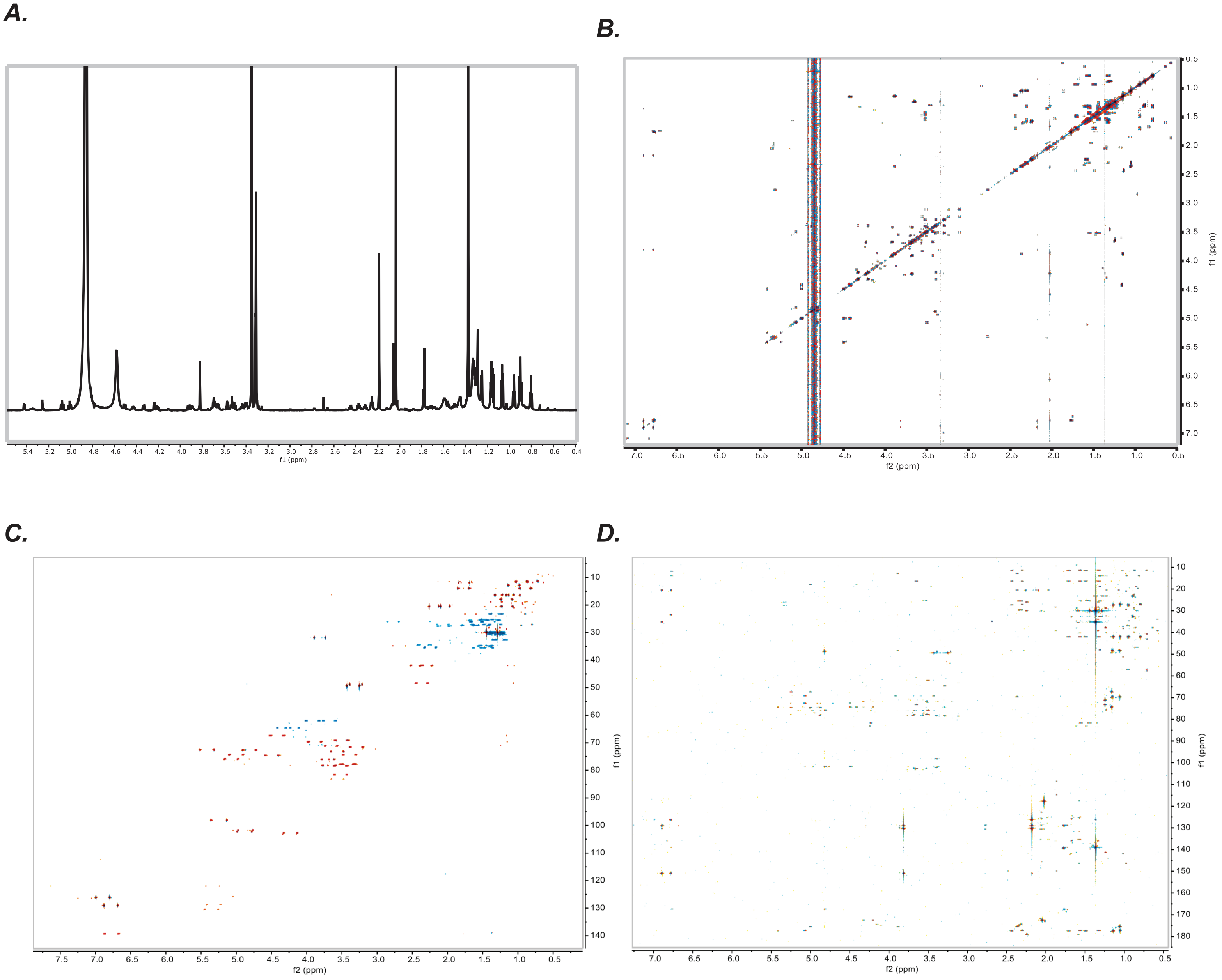

Supplementary Figure 5: NMR spectra of Dichondrin D (800 MHz, CD_3OD) (A) ^1H NMR (B) dqfCOSY (C) HSQC (D) HMBC

Supplementary Figure 6

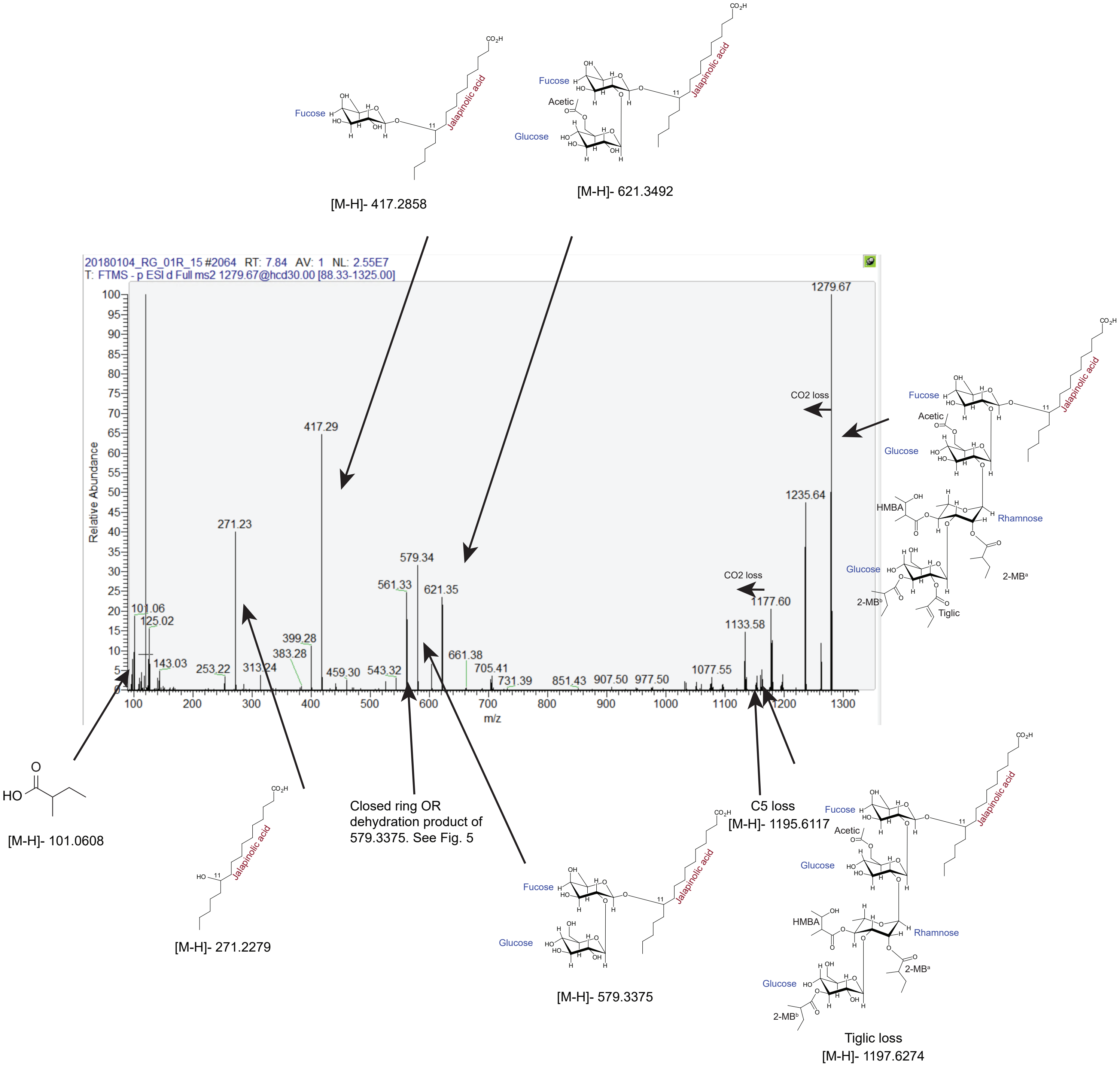

Supplementary Figure 6: Fragmentation pattern of Dichondrin D Structures corresponding to various observed MS/MS fragments in negative ion mode are shown. 561.33 is a dehydration/condensation product of the 579.34 fragment. HMBA: Hydroxymethylbutyric acid, 2-MB: 2-methylbutyric acid.

Supplementary Figure 7

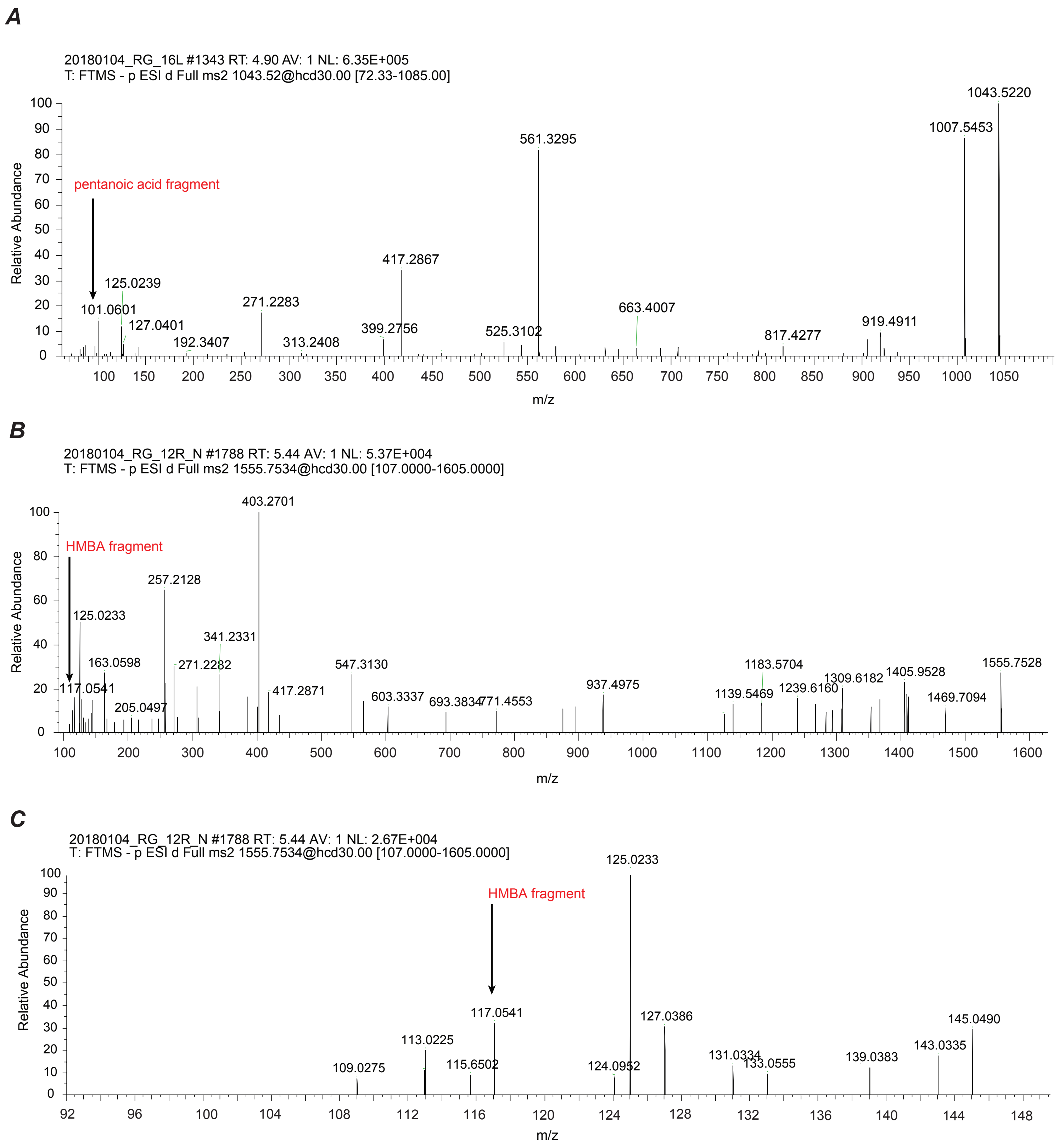

Supplementary Figure 7: Exemplary MS/MS fragments showing the pentanoic acid and hydroxymethylbutyric acid (HMBA) moiety in *Ipomoea* and *Convolvulus* species. Arrows indicate the respective m/z signal of pentanoic acid and HMBA, respectively. (A) RG (m/z 1043.5220) from *Ipomoea tricolor*. (B) RG from *Convolvulus arvensis* Huntley. (C) A zoomed in view of sub-figure B, showing mass range 92 - 150 m/z.

Supplementary Fig. 8

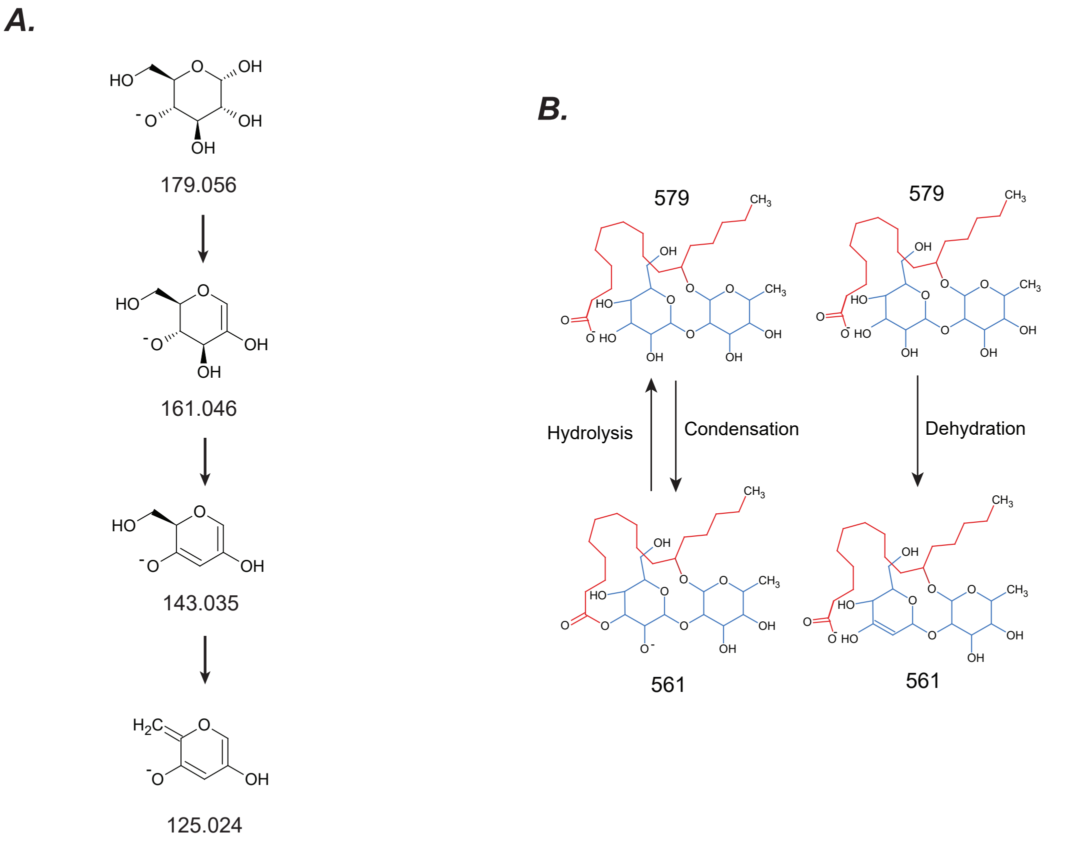

Supplementary Fig. 8: Explaining observed MS/MS fragmentation patterns. All masses denoted are [M-H]- monoisotopic masses of the shown structures (A) Fragmentation pattern of Tricolorin A. (B)Hexose fragmentation producing a 125.024 mass fragment (B) Putative explanations for loss of 18 observed for many neutral losses.
