# Supplementary material for: Illuminating the lineage-specific diversification of resin glycoside acylsugars in the morning glory (Convolvulaceae) family using computational metabolomics
Source: Hortic Res. 2022 Feb 4;9:uhab079. doi: 10.1093/hr/uhab079 (PMC8825387; doi:10.1093/hr/uhab079)
Supplement: Web_Material_uhab079 [file web_material_uhab079.zip › SuppTable1_NMRchemicalshifts.docx.docx]

**Supplementary Table 1:** NMR spectroscopic data (800 MHz) for Dichondrin D

| **Position** | **δ ^13^C [ppm]** | **δ ^1^H [ppm] (*J_HH_*[Hz])** | **HMBC** |
| --- | --- | --- | --- |
| 1 | 179.04 |  |  |
| 2 | 35.56 | 2.24 (t, *J*_2,3_ = 7.6) | 1, 3, 4 |
| 3 | 26.25 | 1.59 | 1, 2, 4 |
| 4 | 30.18 | ~1.3 |  |
| 5-8 | ~30-31 | ~1.25-1.38 |  |
| 9 | 25.48 | 1.35 |  |
| 10 | 34.94 | 1.42-1.50 (m) | 9, 11 |
| 11 | 81.79 | 3.51 (m) | 1′, 9 |
| 12a | 35.67 | 1.44 (m) | 10, 11, 13, 14 |
| 12b |  | 1.55 (m) | 10, 11, 13, 14 |
| 13a | 25.68 | 1.35 |  |
| 13b |  | 1.60 | 12, 15 (weak) |
| 14 | 32.87 | 1.21-1.30 (m) | 12, 15, 16 |
| 15 | 23.46 | 1.31 (m) | 13, 14, 16 |
| 16 | 14.11 | 0.89 (t, *J_15,16_* = 7.3) | 14, 15 |
|  |  |  |  |
|  |  |  |  |
| Fucose |  |  |  |
| 1′ | 102.82 | 4.23 (d, *J_1′,2′_ =* 7.2) | 12, 3′, 5′ |
| 2′ | 78.34 | 3.67 (dd, *J_2′,3′_ =* 9.6) | 1′, 1′′ |
| 3′ | 76.12 | 3.70 (dd, *J_3′,4′_ =* 3.3) |  |
| 4′ | 73.28 | 3.56 (d) | 2′, 3′ |
| 5′ | 71.23 | 3.64 (q, *J_5′,6′_ =* 6.5) | 1′, 4′, 6′ |
| 6′ | 16.53 | 1.24 (d) | 4′, 5′ |
|  |  |  |  |
| Glucose |  |  |  |
| 1′′ | 102.36 | 4.88 (d, *J_1_*_′′_*_,2_*_′′_ = 7.9) | 2′ |
| 2′′ | 77.88 | 3.39 (dd, *J_2_*_′′_*_,3_*_′′_ = 9.3) | 1′′, 1′′′ |
| 3′′ | 78.50 | 3.52 (t, *J_3_*_′′_*_,4_*_′′_ = 9.2) |  |
| 4′′ | 71.74 | 3.29 (t, *J_4_*_′′_*_,5_*_′′_ = 9.1) | 3′′, 5′′, 6′′ |
| 5′′ | 74.48 | 3.38 (ddd, *J_5_*_′′_*_,6_*_′′_*_a_* = 5.5, *J_5_*_′′_*_,6_*_′′_*_b_* = 2.0) |  |
| 6′′a | 64.73 | 4.20 (dd, *J_6_*_′′_*_a,,6_*_′′_*_b_* = 11.6) | 4′′, 5′′, 6′′-acetyl-CO |
| 6′′b |  | 4.33 (dd) | 4′′, 5′′, 6′′-acetyl-CO |
| 6′′-Acetyl-CO | 172.54 |  |  |
| 6′′-Acetyl-CH_3_ | 20.62 | 2.05 | 6′′-acetyl-CO |
|  |  |  |  |
| Rhamnose |  |  |  |
| 1′′′ | 98.29 | 5.24 (d, *J_1_*_′′′_*_,2_*_′′′_ = 2.1) | 2′′, 2′′′, 3′′′, 5′′′ |
| 2′′′ | 72.67 | 5.41 (dd, *J_2_*_′′′_*_,3_*_′′′_ = 3.3) | C=O (2MB^a^) |
| 3′′′ | 74.70 | 4.49 (dd, *J_3_*_′′′_*_,4_*_′′′_ = 9.8) | 1′′′′ |
| 4′′′ | 74.50 | 5.00 (t, *J_4_*_′′′_*_,5_*_′′′_ = 9.8) | C=O (2M3HB) |
| 5′′′ | 67.51 | 4.42 (dq, *J_5_*_′′′_*_,6_*_′′′_ = 6.1) |  |
| 6′′′ | 18.14 | 1.16 (d) | 4′′′, 5′′′ |
|  |  |  |  |
| 2MB^a^ |  |  |  |
| C=O | 177.48 |  |  |
| CH | 42.20 | 2.44 (m) | C=O (2MB^a^) |
| CH-C**H**_3_ | 16.50 | 1.14 (d, *J* = 7.0) | C=O (2MB^a^) |
| C**H**_a_H_b_ | 27.57 | 1.49 (m) | C=O (2MB^a^) |
| CH_a_**H**_b_ |  | 1.72 (m) | C=O (2MB^a^) |
| CH_3_ | 11.76 | 0.95 (t, *J* = 7.5) |  |
|  |  |  |  |
|  |  |  |  |
| 2M3HB |  |  |  |
| C=O | 175.60 |  |  |
| CH | 48.49 | 2.37 (m) | C=O (2M3HB) |
| CH-C**H**_3_ | 16.61 | 1.06 (d, *J* = 7.0) | C=O (2M3HB) |
| C**H**-OH | 69.82 | 3.89 (m) | C=O (2M3HB) |
| CH_3_ | 20.66 | 1.15 (d, *J* = 6.3) |  |
|  |  |  |  |
|  |  |  |  |
|  |  |  |  |
| Glucose |  |  |  |
| 1′′′′ | 101.85 | 4.87 (d, *J_1_*_′′′′_*_,2_*_′′′′_ = 8.0) | 3′′′, 3′′′′, 5′′′′ |
| 2′′′′ | 72.72 | 4.81 (dd, *J_2_*_′′′′_*_,3_*_′′′′_ = 9.8) | 1′′′′, 3′′′′, C=O (tigloyl) |
| 3′′′′ | 76.03 | 5.06 (t, *J_3_*_′′′′_*_,4_*_′′′′_ = 9.6) | 1′′′′ (weak), 2′′′′, 4′′′′, C=O (2MB^b^) |
| 4′′′′ | 69.28 | 3.50 (t, *J_4_*_′′′′_*_,5_*_′′′′_ = 9.6) | 3′′′′, 5′′′′, 6′′′′ |
| 5′′′′ | 78.00 | 3.42 (ddd, *J_5_*_′′′′_*_,6_*_′′′′_*_a_* = 5.6, *J_5_*_′′′′_*_,6_*_′′′′_*_b_* = 2.2) | 4′′′′, 6′′′′ (weak) |
| 6′′′′a | 62.17 | 3.67 (dd, *J_6_*_′′′′a_*_,6_*_′′′′_*_b_* = 11.9) |  |
| 6′′′′b |  | 3.91 (dd) | 4′′′′ |
|  |  |  |  |
| Tigloyl |  |  |  |
| C=O | 167.60 |  |  |
| C=C-CH_3_ | 129.03 |  |  |
| C=C-CH_3_ | 12.10 | 1.77 | C=O (tigloyl), C=C-CH_3_ |
| CH | 139.41 | 6.76 (m) | C=O (tigloyl), CH-CH_3_ , C=C-CH_3_ |
| CH-CH_3_ | 14.14 | 1.77 (d, *J* = 7.4) | C=C-CH_3_ |
|  |  |  |  |
|  |  |  |  |
|  |  |  |  |
|  |  |  |  |
| 2MB^b^ |  |  |  |
| C=O | 177.29 |  |  |
| CH | 42.17 | 2.30 (m) | C=O (2MB^b^) |
| CH-C**H**_3_ | 16.60 | 1.05 (d, *J* = 7.0) | C=O (2MB^b^) |
| C**H**_a_H_b_ | 27.24 | 1.32 (m) | C=O (2MB^b^) |
| CH_a_**H**_b_ |  | 1.56 (m) | C=O (2MB^b^) |
| CH_3_ | 11.56 | 0.79 (t, *J* = 7.5) |  |
|  |  |  |  |
|  |  |  |  |
|  |  |  |  |
|  |  |  |  |
|  |  |  |  |
|  |  |  |  |
